# Validity and reliability of POM-Checker in measuring shoulder range of motion: Protocol for a single center comparative study

**DOI:** 10.1097/MD.0000000000011082

**Published:** 2018-06-22

**Authors:** Hongmin Chu, Seongsu Joo, Jinyoung Kim, Jae Kyoun Kim, Cheolhyun Kim, Jihye Seo, Dae Gill Kang, Ho Sub Lee, Kang-Keyng Sung, Sangkwan Lee

**Affiliations:** a Department of Internal Medicine and Neuroscience, College of Korean Medicine, Wonkwang University, Iksan; b Team Elysium Inc. R&D Center, Seoul; c Clinical Trial Center, Wonkwang University Gwangju Hospital, Gwangju; d Hanbang Cardio-Renal Syndrome Research Center, College of Oriental Medicine, Wonkwang University, Iksan, Jeonbuk; e Internal Medicine and Neuroscience, Jangheung Integrative Medical Hospital, Wonkwang, University, Jangheung, Jeonnam, Republic of Korea.

**Keywords:** Kinect, POM-Checker, protocol, range of motion

## Abstract

**Introduction:**

Assessments of the range of motion (ROM) in human joints have been widely used to evaluate the joint condition. Although maker based motion capture system is the most popular and practical method in the clinical field, there have been limitations such as the relatively long time required in procedure or influence of attached markers on natural movement. Recently, markerless motion capture systems have been actively developed due to their relatively lower cost and convenience for installation. The POM-Checker (Team Elysium Inc., Seoul, Rep of Korea), a markerless motion capture system, was developed with new algorithms to assess the ROM in human joints. However, the measure of the POM-Checker has not been compared with a golden-standard device in evaluating the ROM in the human joints. So we developed a protocol to evaluate the validation and reliability of the POM-Checker in measuring the shoulder ROM. This study will also provide a standard procedure to measure the shoulder ROM with the POM-Checker and 3D motion analysis system simultaneously.

**Methods/design:**

This protocol is for a single institution comparative study to evaluate the validity and reliability of POM-Checker. Six participants will be recruited. We will measure the angles of shoulder abduction and flexion with POM-Checker and 3D motion analysis system simultaneously. The primary outcome is the angles of shoulder abduction and flexion.

**Discussion:**

This will be the first study to analyze the validity and reliability of POM-Checker in measuring shoulder ROM. Although the sample size of this study is small, it may not influence on the results conclusively, because the measures are very precise numerical angles. Furthermore, the angles of shoulder movements will be measured with both devices simultaneously.

**Conclusion:**

The results of the study will be helpful to find out the validity and reliability of a new developed ROM measure device, POM-Checker, by comparison with a golden standard system, 3D motion capture system, in measuring the shoulder ROM. It will also contribute to use of the POM-Checker in measuring the ROMs in many human joints

## 1 Introduction

Assessments of the range of motion (ROM) in human joints have been widely used to evaluate the joint condition and therapeutic effect.^[1]^

Although optical system, marker-based motion capture system, is the most popular and practical method in the clinical field, it has many limitations such like the relatively long time required in procedure or influence of attached markers on natural movement.^[2]^ Recently, many markerless motion capture systems have been developed, because they have many advantages such as convenience in procedure and low purchasing cost.

The Kinect-based devices and markerless motion capture systems have been also developed to be applied to many clinical fields such as rehabilitation for patients with stroke patients^[3,4]^ or sclerosis.^[5]^ However, some Kinect-based devices^[6,7]^ have shown the relatively large range of measure error, 8°to 14°. Therefore, we developed a new Kinect-based device, POM-Checker (Team Elysium Inc., Seoul, Rep of Korea), to assess the ROM in human joints. To improve the error range of ROM measurement with Kinect-based devices, we applied the Kalman-filter-based algorithm instead of Kinect SDK‘s joint recognition function to track joint points. It is expected to improve the accuracy of measurement, because the Kalman filter is an algorithm that uses a series of joint position measurements observed over time. But there is no study conducted to find out the validity and reliability of the POM-Checker in measuring the shoulder ROM. So we developed this protocol to assess the validity and reliability POM-Checker

## 2 Methods and design

### 2.1 Measure systems

The POM-Checker is an isokinetic testing and evaluation system developed by Team Elysium Inc. (Seoul, Korea) which can measure ROM of human joints using the Kinect technology. In POM-Checker, the Kalman-filter-based algorithm was applied instead of Kinect SDK‘s joint recognition function to track joint points to improve the accuracy of the measurement, because the Kalman-filter-based algorithm uses a series of joint position measurements observed over time.

**Figure 1:**
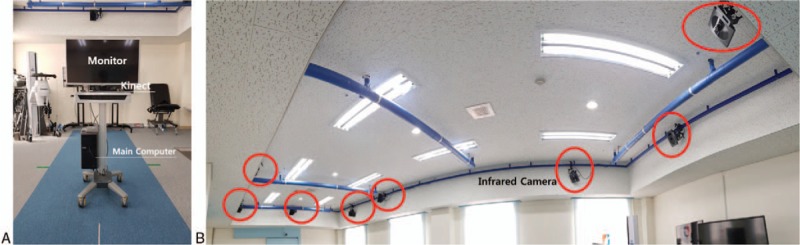
POM-Checker and motion capture system. POM-Checker consists of main computer, monitor, and Kinect. 3D motion capture system has 8 infrared cameras. (A) It is anterior view of POM-Checker. (B) Panorama view of BTS SMART DX-400 at GJWMC. This motion capture system has 8 infrared cameras. Seven cameras are only shown in the picture.

POM-Checker has received conformity assessment (classification code: A30130.01(2)) product license number:18-4334) on isokinetic testing and evaluation systems by Korea Testing Certification (KTC) designated as National Official Professional Testing Research Institute (Fig. 1A).

The 3D motion capture system used in this study is BTS SMART DX-400 (BTS Bioengineering, Italy, Fig. 1B). BTS SMART DX-400 shots100 frames per second. In the global XYZ coordinates, *X*, *Y*, and *Z*-axes are set as anteroposterior directions of the subject, perpendicular to the ground, and direction of right and left of the subject, respectively.

In the shoulder, the definition of abduction or flexions the change of the *Y* values that consist of vector of segments connecting shoulder center and elbow center and vector of the whole body trunk and the *X* values that consist of vector of segments connecting shoulder center and elbow center and vector of the whole body trunk, respectively.

### 2.2 Study design

The study design is a single-center pilot study to assess the validity and reliability of POM-Checker. Participants will be recruited through poster or advertisement in Wonkwang University Korean Medicine Hospitals in Gwangju (GJWKH). Researchers will measure the ROM of shoulder joint by the POM-Checker and 3D motion capture system simultaneously. We will acquire the ROM data from the POM-Checker and 3D motion capture system by simultaneously measuring the shoulder motion and comparing the results of the two devices.

### 2.3 Ethics approval and consent to participate

The pilot study of this protocol (version 1.0) has been approved by ethical approval from the institutional review board (IRB) in GJWKH (WKIRB2017-20, 2018.01.23). For those eligible participants, researchers will give them the detailed information of this study. Participants will provide written informed consent after a clear explanation of the clinical study‘s purposes and characteristics.

Subjects may be required to quit the study in case of serious adverse events and then they will be reported to IRB in GJWKH. In addition, participants may leave this study at any time without any disadvantage or constraint whenever they want to do.

This study will be supervised by the clinical trial center in GJWKH and a clinical research coordinator will coordinate all procedures for participants to improve participants’ adherence to intervention protocols. If there is any change in protocol, it will be approved by IRB again before implementation.

The results will be published on the website of ClinicalTrials.gov in accordance with the CONSORT 2010 Statement and in special journals.

### 2.4 Inclusion and exclusion criteria

Patients will be assigned a patient number, checked whether they meet all the criteria for the enrollment. Inclusion criteria are as follows: participants aged between 18 and 39 years; persons agreeing with participation and providing written informed consent voluntarily after receiving a clear explanation of the clinical study purposes and characteristics; and participants without musculoskeletal disorders in the shoulder joint.

Exclusion criteria are as follows: pregnant women; persons with diseases in nervous immune, respiratory, endocrine or cardiovascular system; persons with tumor or mental illness; persons with musculoskeletal disorders or mechanical abnormalities such as fractures or acute sprains in the shoulder joint; and persons with severe disability or severe pain around the shoulder joint.

### 2.5 Procedure

**Figure 2:**
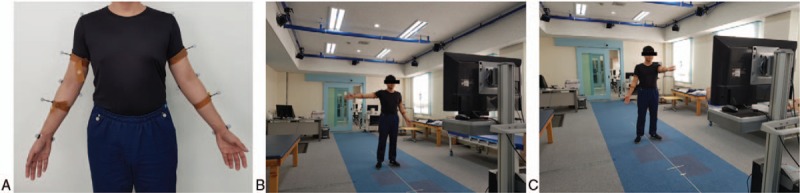
Maker placement and example of trial process. (A) Sixteen silver ball-shaped makers attached to subject. (B) Demonstration of shoulder abduction trial. (C) Demonstration of shoulder flexion trial.

The researchers attach 16 makers on participants‘ upper limb. Upper extremity maker placement was referred to Slavens‘s research.^[8]^ Marker placement (Fig. 2A) are acromion (AC), medial (ME) and lateral epicondyle(LE) of the humerus, styloid process of ulna(SU) and radius (SR), and anterior superior iliac spine (ASIS), middle point of humerus (MH), and middle point of Ulna (MU).

The participants will stand in front of the POM-Checker in the 3D motion capture room and be guided to repeat the shoulder movements, abduction to 90°and flexion to 90°, in order (Fig. 2B and C). After the initial pose sets, we will measure and record the angles of abduction and flexion in the shoulder using the POM-Checker and BTS SMART DX-400 simultaneously. Participants will be required to reset the poses after every motion.

### 2.6 Outcomes

The outcomes are degrees of shoulder ROM, angles of abduction and flexion and equalities of the values measured by the BTS SMART DX-400 and the POM-Checker.

### 2.7 Statistical analysis

Wilcoxon's signed-ranks test will be conducted to assess the differences of the POM-Checker's test-retest consistency and the equalities between the values of the BTS SMART DX-400and POM Checker. All analyses will be performed using the software of statistics analysis system.

### 2.8 Sample size

In the previous study^[6]^ for validity and reliability of a Kinect system in measuring the movement angles in shoulder joint from 10 subjects, standard errors of shoulder abduction and flexion are 2.5° (7.91° as standard deviation, SD) and 4.4° (13.91° as SD), respectively. Given the SD (7.91° or 13.91°), effect size, statistical power, and dropout rate as 10°, 80% and 10%, total sample size can be computed 10 or 31 subjects.

Because 2 computed sample sizes differ considerably, we will recruit 6 subjects to find out the characteristics of measures and obtain the statistical values to compute the sample size for larger following study.

## 3 Discussion

Conventionally, ROM has been widely used to evaluate shoulder function.^[1]^ There are several tools such like still photography,^[9]^ goniometer,^[10]^ and 3D angular measurement^[11]^ for ROM measure in the shoulder joints. However, these tools had limitations such as measure discrepancy between testers.

The marker-based motion capture system has been considered as a standard tool for the ROM measures in human joints. However, it has also limitations that it is required longer time in measure procedure and the attached markers could influence on natural movement.^[2]^ In addition, it has not been popular in clinics because of high cost and big space for installation.

Recently, markerless motion capture systems have been actively developed due to their relatively lower cost and convenience for installation. The POM-Checker (Team Elysium Inc., Seoul, Republic of Korea), a markerless motion capture system, was developed with new algorithms to assess the ROM of human joints.

Therefore, we developed a protocol to evaluate the validation and reliability of the POM-Checker for ROM measure in shoulder joints. Although the trial according to this protocol will be conducted with restrictive inclusion and exclusion criteria, it may have limitations of the small sample size which may lead to bias.

## Acknowledgments

This study was supported by the Traditional Korean Medicine R&D program funded by the Ministry of Health & Welfare through the Korea Health Industry Development Institute (KHIDI) (HI14C0665) and the National Research Foundation of Korea (NRF) grant funded by the Korea Government (MSIP) (No. 2017R1A5A2015805) and Integrative Medicine Research Project, funded by the Ministry of Health & Welfare, Republic of Korea (B0080613000158).

## Author contributions

Contributors: HC, SJ, and JK1 designed and drafted the protocol and manuscript. JK2, CK, and JS examined the inclusion criteria in clinical practice. DK, KS, and LS critically revised the manuscript. SL organized all procedures and revised the protocol. All authors have read and approved the final manuscript.

**Conceptualization:** Hongmin Chu, Seongsu Joo, Jinyoung Kim, Dae Gill Kang, Ho Sub Lee.

**Data curation:** Seongsu Joo, Jinyoung Kim.

**Methodology:** Jihye Seo, SANGKWAN LEE.

**Software:** Jinyoung Kim.

**Supervision:** Cheolhyun Kim.

**Validation:** Jae Kyoun Kim.

**Writing – original draft:** Hongmin Chu, Seongsu Joo, Jinyoung Kim, SANGKWAN LEE.

**Writing – review & editing:** Jae Kyoun Kim, Cheolhyun Kim, Jihye Seo, Dae Gill Kang, Ho Sub Lee, Kang-keyng Sung.
